# CRISPR/Cas9-Mediated Allele-Specific Disruption of a Dominant *COL6A1* Pathogenic Variant Improves Collagen VI Network in Patient Fibroblasts

**DOI:** 10.3390/ijms23084410

**Published:** 2022-04-16

**Authors:** Arístides López-Márquez, Matías Morín, Sergio Fernández-Peñalver, Carmen Badosa, Alejandro Hernández-Delgado, Daniel Natera-de Benito, Carlos Ortez, Andrés Nascimento, Daniel Grinberg, Susanna Balcells, Mónica Roldán, Miguel Ángel Moreno-Pelayo, Cecilia Jiménez-Mallebrera

**Affiliations:** 1 Laboratorio de Investigación Aplicada en Enfermedades Neuromusculares, Unidad de Patología Neuromuscular, Servicio de Neuropediatría, Institut de Recerca Sant Joan de Déu, Santa Rosa 39-57, 08950 Esplugues de Llobregat, Spain; mariacarmen.badosa@sjd.es (C.B.); alejandro.hernandez@uib.es (A.H.-D.); daniel.natera@sjd.es (D.N.-d.B.); carlos.ortez@sjd.es (C.O.); andres.nascimento@sjd.es (A.N.); cecilia.jimenez@sjd.es (C.J.-M.); 2 Centro de Investigaciones Biomédicas en Red de Enfermedades Raras (CIBERER), Av. Monforte de Lemos 3-5, 28029 Madrid, Spain; matmorinro@yahoo.es (M.M.); dgrinberg@ub.edu (D.G.); sbalcells@ub.edu (S.B.); mopelayo@hotmail.com (M.Á.M.-P.); 3 Institut de Recerca Sant Joan de Déu, Santa Rosa 39-57, 08950 Esplugues de Llobregat, Spain; monica.roldan@sjd.es; 4 Servicio de Genética, Hospital Universitario Ramón y Cajal, Instituto Ramón y Cajal de Investigación Sanitaria, Ctra. de Colmenar Viejo Km. 9.100, 28034 Madrid, Spain; sergio.fernandez@hrc.es; 5 Departamento de Genética, Microbiología y Estadística, Facultad de Biología, Institut de Biomedicina de la Universitat de Barcelona (IBUB), Universitat de Barcelona, Av. Diagonal 643, 08028 Barcelona, Spain; 6 Unidad de Microscopia Confocal e Imagen Celular, Servicio de Medicina Genética y Molecular, Institut Pediàtric de Malalties Rares (IPER), Hospital Sant Joan de Déu, Passeig Sant Joan de Deu, 2, 08950 Esplugues de Llobregat, Spain

**Keywords:** congenital muscular dystrophies, collagen VI-related disorders, dominant negative mutations, allele-specific silencing, CRISPR/Cas9, gene editing, *COL6A1*

## Abstract

Collagen VI-related disorders are the second most common congenital muscular dystrophies for which no treatments are presently available. They are mostly caused by dominant-negative pathogenic variants in the genes encoding α chains of collagen VI, a heteromeric network forming collagen; for example, the c.877G>A; p.Gly293Arg *COL6A1* variant, which alters the proper association of the tetramers to form microfibrils. We tested the potential of CRISPR/Cas9-based genome editing to silence or correct (using a donor template) a mutant allele in the dermal fibroblasts of four individuals bearing the c.877G>A pathogenic variant. Evaluation of gene-edited cells by next-generation sequencing revealed that correction of the mutant allele by homologous-directed repair occurred at a frequency lower than 1%. However, the presence of frameshift variants and others that provoked the silencing of the mutant allele were found in >40% of reads, with no effects on the wild-type allele. This was confirmed by droplet digital PCR with allele-specific probes, which revealed a reduction in the expression of the mutant allele. Finally, immunofluorescence analyses revealed a recovery in the collagen VI extracellular matrix. In summary, we demonstrate that CRISPR/Cas9 gene-edition can specifically reverse the pathogenic effects of a dominant negative variant in *COL6A1*.

## 1. Introduction

Collagen VI-related dystrophies (COL6-RDs) are the second most common form of congenital muscular dystrophies. Their severity ranges from relatively mild Bethlem myopathy (OMIM 158810) across intermediate COL6-RD phenotypes to severe Ullrich congenital muscular dystrophy (OMIM 254090) [1,2,3,4,5]. Currently, no effective treatments are available for COL6-RD and the majority of individuals show progressive muscle weakness, distal joint hyperlaxity, contractures of proximal joints and progressive respiratory insufficiency [3,6,7].

COL6-RDs are caused by pathogenic variants in the genes that encode the three major α-chains of collagen VI *COL6A1*, *COL6A2* and *COL6A3*, which are connected by their triple-helical domains (characterized by the Gly-X-Y motif, where Gly is for glycine, X for proline and Y for hydroxyproline or hydroxylysine) twisting around each other to form monomers [8,9]. The monomers assemble into anti-parallel dimers and then tetramers, which are secreted into the extracellular matrix (ECM), where they further assemble with other tetramers to form the characteristic collagen VI microfibrillar matrix [6,10,11].

Although initially described as a recessive disease, dominant variants account for 50–75% of all the pathogenic variants in COL6-RDs [12]. One-third of COL6-RD cases are caused by dominant missense changes that affect glycine residues located within the triple helix domain, and the majority of these variants are clustered at the N-terminal end of the triplex domain. This segment is essential for the correct assembly of collagen VI monomers, likely explaining why these variants have a dominant effect [4,13]. A frequently encountered variant is *COL6A1* c.877G>A; p.Gly293Arg, the most prevalent pathogenic variant in our cohort (see below) and in others [12]. This specific variant alters the structure of the tetramer caused by the substitution of glycine 293 for an arginine at the N-terminal of the triple helix domain. The Gly293Arg substitution introduces folds that prevent proper assembly with other tetramers to form collagen VI microfibrils [13,14]. While the majority (>40%) of individuals with variants causing glycine substitutions have intermediate phenotypes, there is a wide spectrum of disease burdens, including severe (20%) or mild (20%) phenotypes. Remarkably, only 4% of individuals genetically diagnosed with this type of variant in any of the three collagen VI genes are clinically unaffected. Notably, this clinical and phenotypic variability has also been observed in different individuals with the same pathogenic variant [13].

Several lines of evidence indicate that haploinsufficiency of collagen VI per se does not cause disease [8,15,16]. Accordingly, some studies have tested the efficiency of targeting the mutant allele in COL6-RD using RNA interference [17,18] and antisense oligonucleotides (AONs) [14,19]. In these cases, the “treatments” reduced the intracellular levels of collagen VI levels and increased the abundance of collagen VI in the ECM. This suggests that strategies aimed at the specific suppression of the mutated allele, to reduce its dominant-negative effect or to correct the pathogenic variant, would be beneficial for many individuals with COL6-RD. Genome editing based on the CRISPR/Cas9 system offers both possibilities. Specifically, the CRISPR system (clustered regularly interspaced palindromic repeats) allows the targeting of a CRISPR-associated endonuclease (Cas) to a specific DNA sequence with high precision to generate double-stranded cleavage [20,21]. The cell would then repair this damage using one of two alternative mechanisms: non-homologous end joining (NHEJ) and homology-directed repair (HDR). In the former mechanism, the DNA ends join together in a non-homologous manner, resulting in nucleotide insertion or deletion (INDEL) variants at the cleavage site, a proportion of which will cause inactivation of the allele. In the latter mechanism, the cleavage site is repaired by homologous recombination if a DNA template with the desired sequence is added to the system [22,23,24]. A substantial body of literature describes the applications of CRISPR/Cas9 for the treatment of human hereditary diseases, including its potential and versatility as a sophisticated tool for dominant heterozygous variants in human cells [25].

Here, we describe the results of CRISPR/Cas9-mediated editing in dermal fibroblasts from four different individuals all presenting with the dominant-negative pathogenic variant *COL6A1* c.877G>A; p.Gly293Arg. Using next-generation sequencing (NGS) analysis to survey the allelic variability generated in the four fibroblast cell pools, we found that the repair of the mutant allele by HDR occurred at a frequency of <1%. A large proportion of the editing events that had occurred were due to out-of-frame INDELs that would potentially silence the mutant allele. We found that the silencing event occurred with high specificity, as the wild-type allele was virtually intact, with no undesired edits. Specific silencing of the mutant allele was confirmed at the transcriptional level, which resulted in an increase in collagen VI secretion and an improvement in the collagen VI pattern in the ECM. Overall, the present study demonstrates the great potential of CRISPR/Cas9 technology for the treatment of neuromuscular diseases caused by dominant-negative pathogenic variants.

## 2. Results

### 2.1. Design and Analysis of crRNAs Targeting Exon 10 of COL6A1

**Figure 1:**
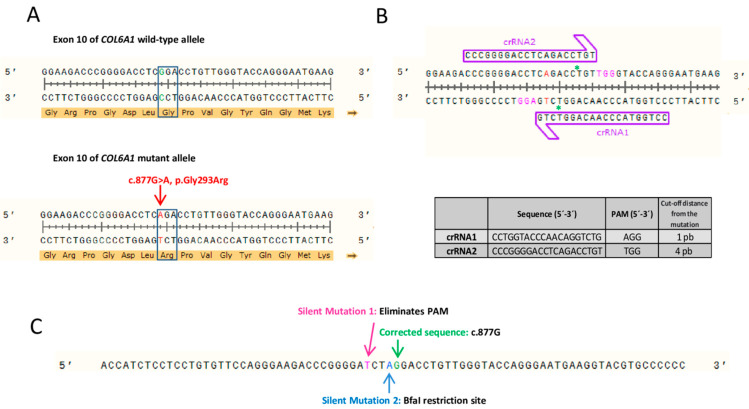
Strategy for CRISPR/Cas9 editing of c.877G>A variant in exon 10 of *COL6A1*. (**A**) The c.877G>A, p.Gly293Arg dominant-negative pathogenic variant is heterozygous in the cohort of individuals studied here. (**B**) Schematic representation of the experimental design of the two RNA guides (crRNA1 and crRNA2). Mutant adenine is shown in red. Protospacer adjacent motif (PAM) sequences are highlighted in pink for each crRNA. Green asterisks (*) represent the predicted Cas9 cleavage site for each crRNA. The table shows the sequence of each of the crRNAs, their corresponding PAM and the nucleotides that separate the Cas9 cut-off point of the variant. (**C**) Sequence of the single-stranded DNA template delivered to fibroblasts to correct the pathogenic variant by homology-directed repair. In addition to the wild-type guanine nucleotide at position c.877, it contains two extra silent changes to eliminate the PAM and generate a restriction site for the BfaI.

We designed two CRISPR RNA guides (crRNAs) to correct the dominant-negative variant in exon 10 (*COL6A1* c.877G>A; p.Gly293Arg) (Figure 1A). Several putative crRNAs were identified using the Breaking-Cas web-tool [26], and two were chosen based on their greater proximity to the pathogenic variant, the score that the software provided and the fewest potential off-targets. In both cases, the possible off-target that was recorded with greater probability was the same exon 10 region of the wild-type *COL6A1* allele. However, according to the web tool, the probability that the guides would bind and induce cleavage of this allele was very low. The remaining potential off-targets identified by Breaking-Cas had a probability of <2%. The target sites of the crRNAs were located in close proximity (1- and 4-bp upstream) to the target pathogenic variant (Figure 1B). No other sequence within the human genome showed 100% identity with the crRNA target sites.

We also designed an 80-nucleotide-long ssDNA donor template containing the wild-type sequence of *COL6A1* exon 10 (a guanine at position c.877). Two additional silent changes were introduced into the ssDNA donor-template sequence. One change, c.873C>T, was added to eliminate the protospacer adjacent motif (PAM) sequence and avoid re-editing events in the same locus. The other one, c.876C>A, was included to generate a BfaI restriction site (Figure 1C). This restriction site would only be present in cells that had undergone editing and incorporated the donor DNA template by homologous recombination. A restriction fragment length polymorphism (RFLP) assay using the BfaI enzyme was used to identify cells incorporating the donor template.

### 2.2. CRISPR/Cas9 Transfection and Next Generation Sequencing of Fibroblasts

Skin fibroblast cultures from four individuals were transfected with the ribonucleoprotein complex (RNP) formed by the Cas9 endonuclease and the duplex RNA from the union of each of the crRNAs and a fluorescently labeled transactivating crRNA (tracrRNA-ATTO550), together with the ssDNA template. The RNPs formed by each of the two guides (crRNA1 and crRNA2) were transfected individually and in parallel in each experiment. We also performed a reverse transfection in which the fibroblast suspension was added to the mix containing the different CRISPR system components and the lipofection agent. Fluorescence-activated cell sorting (FACS) was performed using the tracrRNA-ATTO550 fluorophore to measure transfection efficiency and to select the transfected fibroblasts. Results showed that transfection of crRNA1 or crRNA2 resulted in >80% of the cells positive for ATTO550, indicating the incorporation of the CRISPR system (Supplementary Figure S1). This high transfection efficiency was expected to result in a high editing rate.

**Figure 2:**
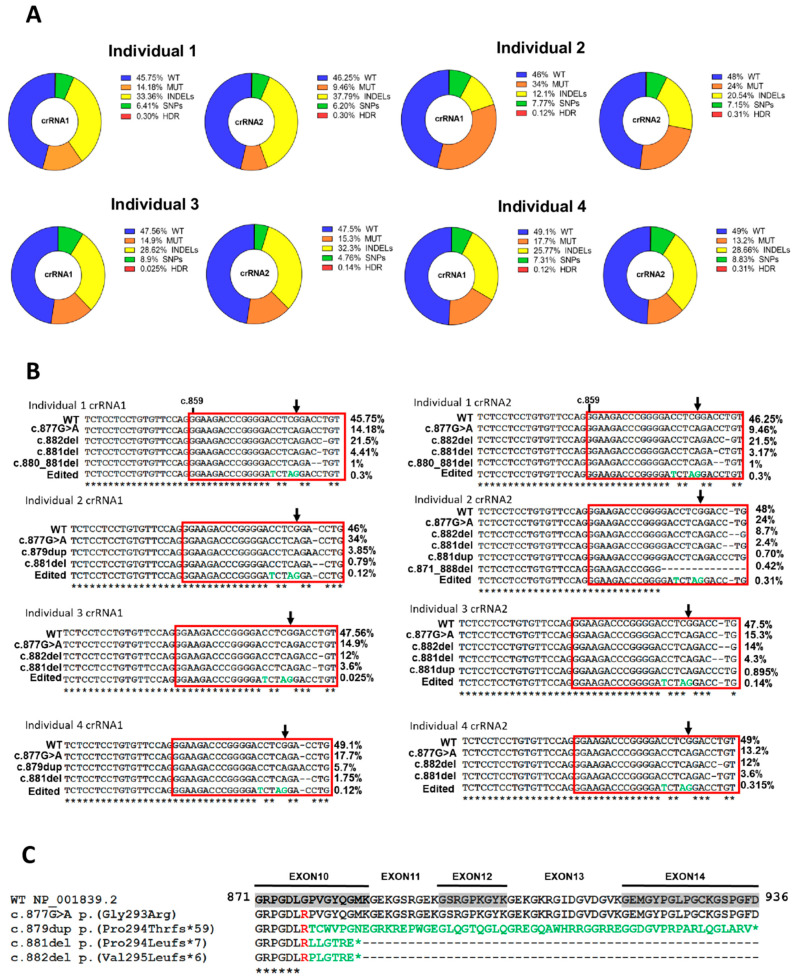
Analysis of the allelic variability in edited patient-derived fibroblasts. (**A**) Wild-type (WT), mutant (MUT), insertions and deletions (INDELs), single nucleotide polymorphisms (SNPs) and homology-directed repair (HDR) allele percentages after analysis of edited fibroblasts of the four individuals by next-generation sequencing. (**B**) Alignment of the most frequent allelic variants at the cDNA level in each of the fibroblasts of the individuals edited with crRNA1 or crRNA2. The changes introduced in the ssDNA template are represented in green in the variant allele resulting from the HDR. (**C**) Alignment at the protein level of the WT sequence encoded by exons 10 to 14 of the mutant variants associated with the most frequent alleles. Arginine present in the pathogenic allele is shown in red and aberrant tails up to the premature stop codon (*) in green.

Genomic DNA reads from the four cell populations transfected with both cRNA1 and crRNA2 were analyzed using Mosaic Finder [27,28,29,30] to evaluate the editing success and quantify the allelic variability. Correction of the double-strand break (DSB) in the DNA induced by CRISPR/Cas9 and mediated by HDR occurred at a very low frequency, being less than 0.5% in most of the allelic variants (Figure 2A). This occurred in all individual fibroblast cultures, both with crRNA1 and crRNA2. We then examined the expected high allelic variability associated with NHEJ repair using Mosaic Finder to survey the repertoire of allelic variants generated in fibroblasts after CRISPR/Cas9 editing. The presence of the wild-type COL6A1 allele was almost unaltered in all individual’s cells, indicating that both crRNA1 and crRNA2 have high specificity for the mutant allele (Figure 2A). The percentage of reads showing the edited wild-type allele with both crRNA1 and crRNA2 was, in most cases, very low. This is relevant, as the main aim of the approach is to induce the specific silencing of the mutant allele without affecting the wild-type allele, which recovers the normal formation of the collagen VI matrix [18,19]. Notably, the presence of the mutant allele was markedly reduced from 50% to 10% in most cases (Figure 2A), demonstrating the high efficiency of the CRISPR/Cas9 system to specifically target the mutant allele. We expected that the correction of the DSB in the mutant allele by NHEJ would lead to gene silencing in a high proportion of cases. We detected a range of allelic single nucleotide variants found with variable frequency; some of these might give rise to nonsense variants, ultimately producing silencing. Another group of allelic variants detected in all edited fibroblasts was a highly varied and diverse set of INDELs (Figure 2A), which were related to the fibroblast source and the crRNA used and included the variants c.881del, c.882del and c.879dup, among others (Figure 2B). Most of these INDELs caused translation frameshifts, generating premature stop codons and, likely, functional abrogation of the mutant protein. Multiple protein sequence alignments of the wild-type amino acid sequence encoded by exons 10 to 14 and the mutant proteins associated with the most frequent alleles are shown in Figure 2C.

Nucleotides that were most frequently deleted were those nearest the Cas9 cleavage site, specifically, where Cas9 produces the DSB with each of the RNA guides: c.878 for crRNA1 and c.881 for crRNA2 (Supplementary Figure S2). These results support the high specificity of both crRNA guides to bind to the correct site of the mutant allele and induce DSB.

### 2.3. NHEJ-Mediated COL6A1 Editing of Patient Fibroblasts Reduces the RNA Levels of the Mutant Allele

**Figure 3:**
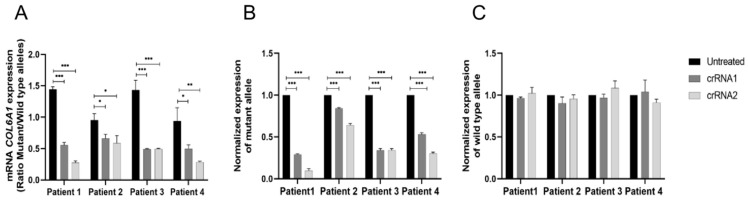
Transcriptional silencing of the mutant allele in patient-derived fibroblasts. Measurement of mRNA levels of the mutant and wild-type alleles of COL6A1 by allele-specific digital-droplet PCR in individuals’ fibroblasts. (**A**) Ratio of the expression of mutant versus wild-type alleles in fibroblasts untreated or edited with crRNA1 or crRNA2. (**B**) Normalized expression of the mutant allele in the edited fibroblasts. (**C**) Normalized expression of the wild-type allele in the edited fibroblasts. Data are presented as mean ± SD (*n* = 3). Student’s *t* test was used to compare the data (* *p* < 0.05; ** *p* < 0.01; *** *p* < 0.001).

To verify that the allelic variants identified by NGS did indeed result in the specific silencing of the mutant allele, we next evaluated the allele-specific expression of *COL6A1*. As the mutant and wild-type alleles differ by only a single nucleotide, we used ddPCR to differentiate between the specific expression of each allele using specific probes for wild-type or mutant cDNA. The results showed that the expression ratio of the mutant versus wild-type allele was markedly lower (~0.3) in edited fibroblasts than in unedited fibroblasts (1–1.5) (Figure 3A). Examination of the normalized expression of the mutant allele showed that it was reduced in all cases, reaching >80% in individual 1 fibroblasts treated with crRNA2 (Figure 3B). This indicates the specific silencing of the mutant allele, as the wild-type allele was expressed at normal levels compared with the unedited fibroblasts (Figure 3C). Overall, crRNA2 appeared to induce greater silencing than crRNA1. Of note, silencing was less evident in fibroblasts from individual 2, which correlates with the NGS analysis of the edited allele analyzed by Mosaic Finder. Despite the evident variability between the individual fibroblast populations, the specific transcriptional silencing of the mutant allele was confirmed.

It has previously been described that partial silencing of other *COL6* dominant-negative pathogenic variants significantly improves collagen VI assembly and ECM organization [18,19,31]. We thus studied whether silencing of the mutant *COL6A1* c.877G>A allele would translate into a recovery of fibroblast function to form a collagen VI matrix, which is the final therapeutic objective of this approach.

### 2.4. Silencing of the Mutant Allele Partially Restores Collagen VI Intensity and Microfibrillar Structure in the Extracellular Matrix

**Figure 4:**
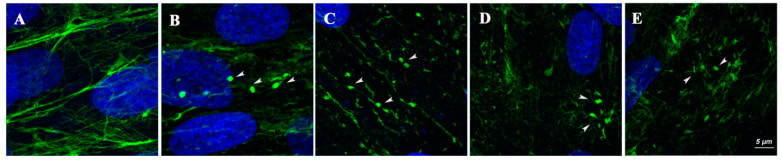
High-resolution images of the collagen VI extracellular matrix. Representative images (blue = nuclei, green = collagen VI matrix) of fibroblasts from healthy controls (**A**) and from the four individuals (1–4, **B**–**E**, respectively) showing collagen VI globular speckles (arrows), often connected by thin fibrils, a diffuse staining pattern and general fragmentation of collagen VI fibrils in individual samples compared with an organized network of fibers from a healthy control.

**Figure 5:**
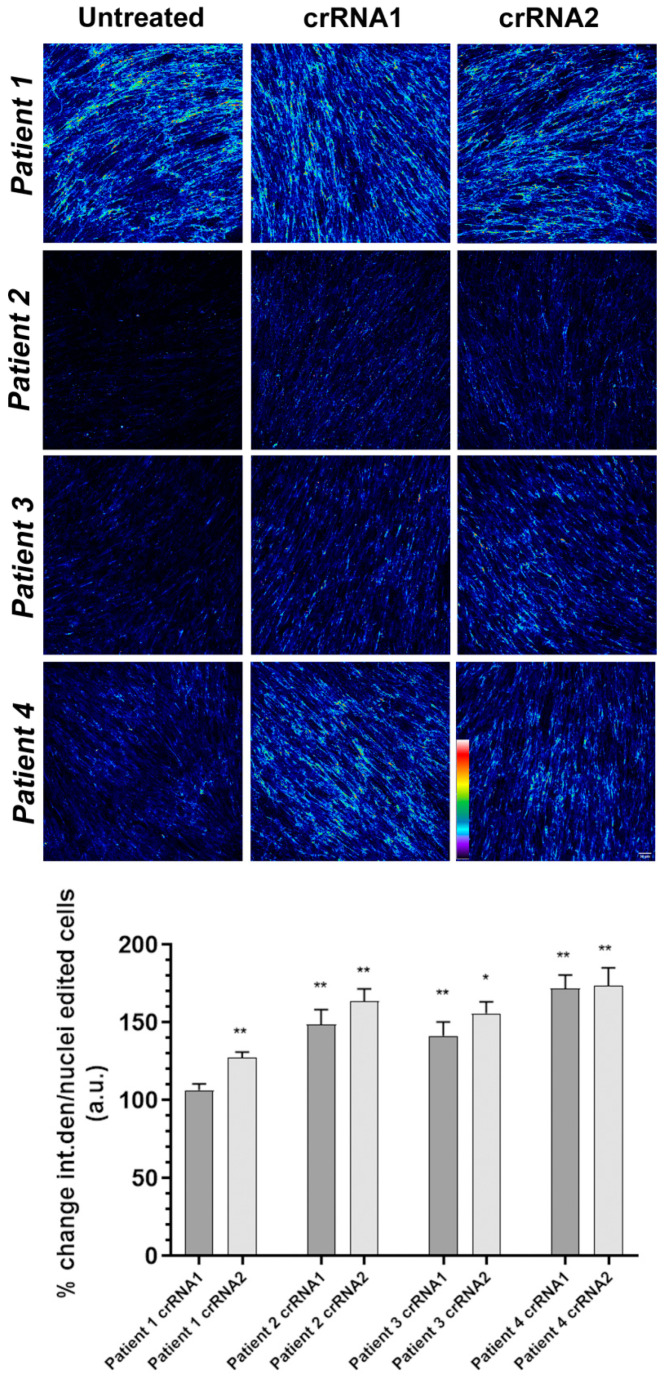
Pseudocolor confocal representative images of collagen VI. Pseudocolor confocal projections of collagen VI extracellular matrix derived from individuals’ fibroblasts before or after treatment with the corresponding cRNA guides. An increase in fluorescence intensity was observed after treatment. The pseudocolor scale is shown on the bottom right. Warm colors such as white and red represent maximum intensities, whereas cold colors like blue are representative of low intensities. Scale bar = 50 µm. The lower graph corresponds to quantification of the intensity of collagen VI in the extracellular matrix of unedited and edited fibroblasts of four individuals (1–4) with crRNA1 and crRNA2 represented as the percentage of change ± SD with respect to the untreated individual. Data were analyzed by the Wilcoxon-paired test (* *p* < 0.05; ** *p* < 0.01).

**Figure 6:**
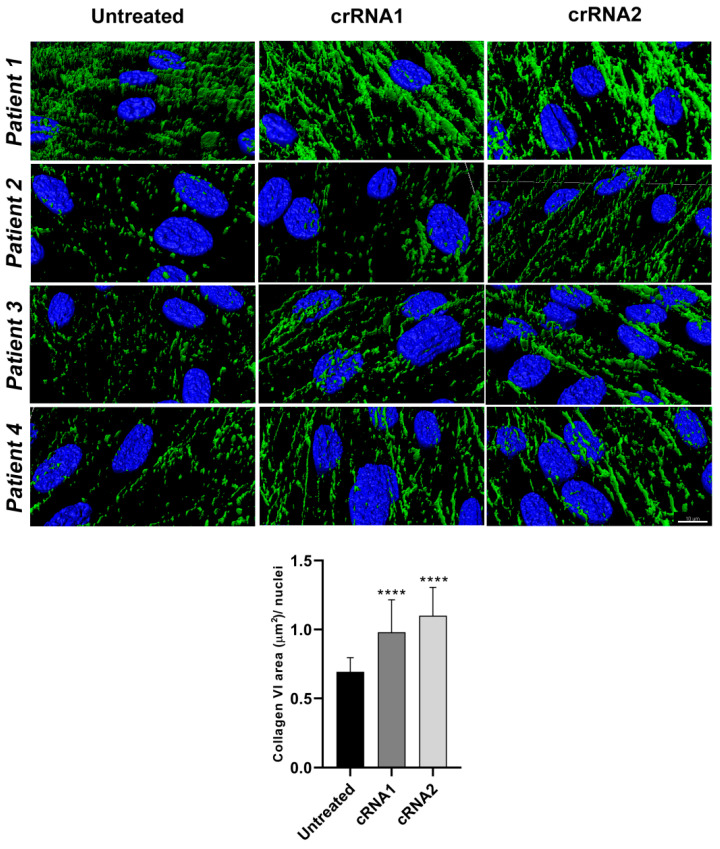
High-resolution 3D rendering images of the collagen VI extracellular matrix before and after editing. Representative images of fibroblasts from four individuals (blue = nuclei, green = collagen VI matrix) before and after treatment with cRNA1 and cRNA2, showing the fragmentation of collagen VI fibrils in untreated cells and their recovery after editing. Images of the collagen VI matrix (green) show few and very thin fibers (or spots/globular aggregates) in the untreated fibroblasts. An interconnected network of collagen VI was observed after treatment. Scale bar = 10 µm. The lower graph corresponds to the mean ± SD of the quantification of the area of collagen VI in the ECM of unedited and edited fibroblasts of individuals 1–4 with crRNA1 and crRNA2. Data were analyzed by the Wilcoxon-paired test (**** *p* < 0.0001).

To evaluate the effect of silencing the *COL6A1* mutated allele on the collagen VI microfibrillar ECM, we performed immunofluorescence and confocal super-resolution microscopy (Figure 4, Figure 5 and Figure 6) following previously reported methods [32,33].

With the exception of fibroblasts from individual 1, all primary fibroblasts showed a basal (prior to treatment) reduction in the intensity of the collagen VI signal relative to control cells, which ranged from 30% to 60% of the intensity levels calculated in the corresponding control fibroblasts. Additionally, in all cases, we observed qualitative differences between control and mutant cells, such as collagen VI-positive globular speckles between the cells often connected by thin fibrils, a diffuse staining pattern and discontinuous and fragmented collagen VI fibrils (Figure 4), as previously reported in individuals with this type of variant [12,13].

We measured the intensity of the collagen VI signal in each of the four fibroblast populations before and after CRISPR/Cas editing. The intensity analyzed with Fiji/ImageJ and represented by the parameter “integrated density” was normalized by the number of nuclei in each of the ten fields for each condition. Representative images of each fibroblast population, untreated or treated with cRNA1 or cRNA2, are shown in Figure 5. A pseudocolor palette was added to visualize the different degrees of intensity of the collagen VI positive signal in each image. Warm colors such as white and red represent maximum intensities, whereas cold colors like blue are representative of low intensities.

The percentage of recovery of the collagen VI signal (average integrated density/nuclei) relative to untreated cells (considered 100%) is shown in Figure 5 (intensity histograms). Overall, we observed a significant increase in the intensity of both cRNAs. In individual 1, the increase in collagen VI intensity was significant only with cRNA2. The percentage increase in intensity was greater in the fibroblasts from individuals who showed a more marked reduction in the intensity of collagen VI at baseline (individuals 2, 3 and 4), particularly in individual 4. These results indicate that the effect of silencing the mutated *COL6A1* allele on the recovery of collagen VI detectable in the ECM varies between samples and might be related to the baseline reduction of collagen VI before treatment.

To better visualize the qualitative changes in the morphology of the collagen VI microfibrils, we performed super-resolution microscopy on cells labeled for collagen VI before and after CRISPR/Cas editing and obtained 3D rendering images (Figure 6 and Supplementary Video S1–S4). In fibroblasts from individual 1, all collagen VI-positive fibers were very fragmented before treatment, whereas after treatment (with either cRNA), the collagen VI-positive fibers connected and formed thick fibers similar to that observed in control cells. Furthermore, only a few abnormal globular collagen VI speckles were observed after cRNA1 or cRNA2 editing. In unedited fibroblasts from individuals 2 and 3, collagen VI microfibrils were scarce (in keeping with the intensity analyses described earlier) and appeared fragmented and composed mainly of isolated speckles. After editing, we observed an increase in collagen VI continuous microfibrils, which appeared more similar to those of control cells, albeit at a lower amount. In both cases, the recovery appeared more pronounced with cRNA2 than with cRNA1. In fibroblasts from individual 4, although the basal collagen VI microfibrillar network appeared more structured than in individuals 2 and 3, there was a visible improvement in the morphology and continuity of the collagen VI fibrils relative to untreated cells after treatment with either cRNA1 or cRNA2. To obtain a quantitative indication of the fragmentation of the collagen VI microfibrillar network and its recovery after treatment, we measured the area of the fibrils in the super-resolution images. The data were normalized by the nuclei in each field. The results showed significant differences between untreated cells and treated cells with either guide in all cases (Figure 6), depicted as the average area of collagen VI fibrils in untreated and treated fibroblasts.

## 3. Discussion

COL6-RDs are a group of neuromuscular conditions caused by pathogenic variants in the three genes that encode collagen VI subunits. More than half of all individuals, including many with the most severe phenotype, present with dominant-negative pathogenic variants [3]. No effective treatments are currently available, although recent experimental studies have proposed silencing of the pathogenic mutant allele as a therapeutic strategy. This is feasible because the pathogenic effect of the variant can be reversed by increasing the proportion of wild-type collagen VI chains relative to mutant chains [18]. Different approaches have been used to test this, such as those based on gapmer AONs or siRNAs [18,19,31]. In this context, the emergence of CRISPR/Cas9 technology has brought a vast range of new possibilities to the field of genetically based diseases, including neuromuscular diseases [25,34].

In the present study, we used two non-exclusive strategies in parallel to boost the prevalence of the wild-type *COL6A1* allele in fibroblasts of individuals harboring the recurrent dominant-negative *COL6A1* c.877G>A; p.Gly293Arg pathogenic variant, which is one of the most common variants in COL6-RD [4]. One strategy was based on the HDR of the variant using a ssDNA template that contained the wild-type guanine in the corresponding residue of exon 10 of *COL6A1*. In the event that the homologous recombination-based repair of DSB induced by Cas9 was not successful, we studied whether the allelic variants obtained led to the specific silencing of the mutant allele. In this sense, it was possible to confirm the allelic variability occurring in the pools of the four edited fibroblast populations from individuals using NGS and bioinformatic analysis. In all cases, we corroborated that HDR occurred at a very low frequency. It has been described that the frequency of HDR is dependent on the cell type that is being edited [35]. For fibroblasts, this rate has been reported using CRISPR and other nuclease-based gene editing methods [36,37,38]. Indeed, this low frequency remains a major stumbling block to the use of this technique as a therapeutic strategy in vivo. In studies of animal models, HDR editing efficiency is generally between 0.1% and 6.5% [39,40,41,42,43]. Various strategies have been proposed to meet the challenge of increasing the rate of HDR [44,45,46]. For example, the delivery of the DNA template in an adeno-associated viral vector was found to increase the frequency of correction of a *COL7A1* pathogenic variant by HDR by up to 50% in keratinocytes from individuals with epidermolysis bullosa [47,48]. In the context of CRISPR, new strategies under development might be a useful alternative for addressing the correction of point variants, including the use of base editors [49,50] or prime editing [51]. These technologies are expected to increase the editing rate, since they are not dependent on inducing DSBs in the DNA and do not rely on HDR. Notably, the correction of point variants with these gene-editing techniques is independent of the cell cycle, so they should be useful in highly differentiated cell types with a low rate of division [39]. Promising advances have recently been made in the in vitro use of these techniques in fibroblasts [52,53], which are the target cell type of the pathology discussed here. The results of the present study suggest that NHEJ-mediated repair is equally valid, as silencing (even partial) of the mutant allele was effective in improving the collagen VI microfibrillar network in the ECM, as reported [18]. In this context, it is crucial that silencing is as specific for the mutant allele as possible and preserves the expression of the wild-type allele, increasing the proportion of wild-type collagen VI transcripts and maintaining the stoichiometric ratio for collagen VI assembly [6]. We confirmed this by quantifying the expression of *COL6A1* allele-specific mRNAs, which revealed a high specificity of the system for the pathogenic allele, as almost no editing was observed in the wild-type allele in most samples. It is therefore likely that so-called off-targets will not be a concern, as the wild-type allele was predicted to be the potential off-target with a higher probability of being edited. Furthermore, while off-target editing remains an issue to be considered, it does not represent a real problem in most cases, as previously proposed [54,55] and demonstrated here. The small differences observed between individuals might be due to individual genetic backgrounds, and it will be important to investigate the potential variability between individuals for future uses of this approach as a therapeutic strategy.

Morphologically, we observed that edited cells overall showed an improvement in the pattern of collagen VI microfibrils, which became more continuous and interconnected, similar to control fibroblasts, and with an average increase in the area corresponding to collagen VI positive fibrils. This was particularly evident in the 3D super-resolution reconstructions. In addition, the collagen VI-positive aggregates often seen in fibroblast cultures from individuals with the Gly293Arg pathogenic variant became almost unnoticeable. Moreover, the edited cells showed an increase in the intensity of collagen VI, which was significant in all cases with cRNA2.

It is also important to discuss the variability between individuals. It is known that individuals with COL6-RD who share this same variant and those with other glycine substitutions in the neighboring triple helical region can present with a severe, intermediate or mild phenotype and also show differences in the severity of the collagen VI reduction and morphological abnormalities in fibroblast cultures, meaning that genotype-phenotype correlations are not always strong [12,13]. Variability is likely due to the effect of currently unknown disease modifiers at the genetic and biochemical levels likely related to ECM components [56,57,58]. This variability was also observed in the group of individuals studied here. With the exception of individual 1, all four individuals showed a decrease in basal (pre-treatment) collagen VI fluorescence intensity in fibroblasts, with individuals 2 and 3 showing the most severe reduction followed by individual 4. Similarly, fibroblasts also varied with regards to the efficacy of the editing, which requires further investigation and indicates that a specific strategy of gene correction may not be appropriate for all individuals even if they carry the same pathogenic variant. Previous studies using siRNA or AONs to correct specific pathogenic variants in collagen VI genes [18,19] included fewer individuals, and likely this inherent variability in the effect of the therapeutic approach was not as apparent as in our study with four individuals.

Our results support the use of this CRISPR/Cas strategy as a therapeutic approach for individuals with COL6-RD, although many complex aspects remain to be resolved, such as the delivery system to the target organs. The use of gold nanoparticles has been proposed to deliver CRISPR/Cas components in vivo [59], allowing the association of cargo proteins, for example, to engage specific cell receptors on the surface of the fibroblasts to increase the specificity to this specific cell type. This is an advantage when compared with other systems, such as viral vectors, where the control of cell type specificity is more challenging. On the other hand, the skeletal muscles are the most affected organs in COL6-RD, although fibroblasts are the target cell type. It is feasible to think that both local and systemic delivery should be tested to deliver the CRISPR machinery in vivo, as has been done for other neuromuscular pathologies, such as Duchenne muscular dystrophy [34,60]. Another question is whether the already deposited mutant collagen VI would continue to exert its effect on the non-mutated or corrected collagen VI. However, considering the turnover of collagen VI in the extracellular matrix, it would be expected that the aberrant collagen VI would be progressively replaced by functional collagen VI produced by the edited cells. These issues should be addressed in future in vivo studies, preferably through the use of animal models. In that sense and given that the crRNA guides and CRISPR machinery recognize a particular locus, a specific murine model for this particular mutation will be necessary that recapitulates the phenotype of the disease. 

## 4. Materials and Methods

### 4.1. Human Fibroblasts

Human COL6-RD fibroblasts were isolated from the skin biopsies of four individuals. Control fibroblasts were obtained from HSJD Biobank, Barcelona, Spain. These individuals have been previously described [4]. Individual 3 had Bethlem myopathy and individuals 1, 2 and 4 showed an intermediate phenotype. Fibroblasts were grown in Dulbecco’s modified Eagle’s medium with 10% fetal bovine serum (FBS), 1 × penicillin/streptomycin, and 1 × glutamine (all from Gibco, Waltham, MA, USA) at 37 °C with 5% CO_2_.

### 4.2. crRNAs and ssDNA Donor Template Design

Two crRNAs (crRNA1 and crRNA2) were designed adjacent to the PAM site of the *COL6A1* locus using the Breaking-Cas web tool [61]. The 20-nt-long specific sequences for targeting the COL6A1 gene were 5′-CCTGGTACCCAACAGGTCTG-3′ (crRNA1) and 5′-CCCGGGGACCTCAGACCTGT-3′ (crRNA2). An 80-nt-long ssDNA donor template was designed containing the wild-type *COL6A1* sequence and two silent changes to eliminate the PAM sequence and to generate a restriction site for BfaI. The sequence of this ssDNA oligo was as follows: 5′-ACCATCTCCTCCTGTGTTCCAGGGAAGACCCGGGGATCTAGGACCTGTTGGGTACCAGGGAATGAAGGTACGTGCCCCCC-3´. These reagents and the Alt-R tracrRNA-ATTO550 were synthesized using Integrated DNA Technologies (IDT, Coralville, IA, USA).

### 4.3. Cell transfection

crRNAs and tracrRNA-ATTO550 reagents were resuspended at 100 μM in 1 × Tris-EDTA (TE buffer), pH 8, solution (IDT). Lipofectamine™ CRISPRMax™ (ThermoFisher Scientific Waltham, MA, USA) was used for the transfection of RNP complexes. First, guide RNA complexes were formed by mixing the crRNA and tracrRNA-ATTO550 in equal molar amounts in IDT Duplex Buffer (30 mM HEPES, pH 7.5, 100 mM potassium acetate) at 1 μM by heating the oligonucleotides to 95 °C and gradually cooling to room temperature. For RNP preparation, an RNP-crRNA mix was prepared in OptiMEM^®^ (Gibco, Waltham, MA, USA), containing the RNA duplex (1 µM), HiFi Cas9 (1 µM) and HiFi Cas9 Plus Reagent (both from ThermoFisher Scientific, Waltham, MA, USA). The solution was incubated for 5 min at room temperature. Subsequently, the ssDNAs (100 µM) were diluted to a concentration of 1 µM in TE Buffer. Subsequently, a transfection mix of each guide was prepared in duplicate in OptiMEM^®^ as follows: 1 µL ssDNA at 1 µM, 200 µL of RNP at 1 µM and 9.6 µL Lipofectamine™ CRISPRMax™. The final mixture was incubated for 20 min at room temperature. Next, a cell suspension of 400,000 cells/mL in DMEM without antibiotics was prepared. For each volume (800 µL in a 12-multiwell plate) of cell suspension assigned to each guide, 400 µL of the corresponding RNP-ssDNA mixture was added. Finally, CRISPR-transfected fibroblasts were cultured for 24 h at 37 °C with 5% CO_2_ before analysis.

### 4.4. Fluorescence-Activated Cell Sorting 

FACS analysis of CRISPR-transfected fibroblasts was performed on the BD FACS Aria™ Fusion flow cytometer platform (Becton Dickinson, San José, CA, USA). Cells were harvested 24 h after transfection and a cell suspension 1 × 106 cells/mL was prepared in 500 µL of PBS plus 2% FBS. Selected ATTO550-positive fibroblast cells were cultured in 60-mm culture dishes at 37 °C with 5% CO_2_ in the culture medium described above.

### 4.5. Genomic DNA Extraction

Genomic DNA of sorted CRISPR-transfected fibroblasts was prepared using the DNAeasy Tissue and Blood kit (Qiagen, Hilden, Germany). DNA concentration was measured by spectrophotometry on an ND-1000 UV Spectrophotometer (Nanodrop Technologies, Wilmington, DE, USA). Samples were stored at −20 °C.

### 4.6. RNA Extraction and Retrotranscription

Extraction and purification of total RNA from cell cultures were performed with the RNeasy^®^ Fibrous Tissue Mini Extraction Kit (Qiagen, Hilden, Germany). RNA concentration was measured as for DNA. Samples were stored at −80 °C. For reverse transcription, equal amounts of RNA (1 μg) were used and added to an M-MLV reverse transcriptase reaction mix (Promega, Madison, WI, USA).

### 4.7. Characterization of Gene-Edited Allelic Variants

DNA fragments covering the target region for the two designed crRNAs (crRNA 1 and 2) at exon 10 of *COL6A1* (NM_001848.3; LRG_475) were PCR-amplified using customized primers that included the adapters (upper case) for NGS sequencing followed by the gene-specific sequence (lower case): COL6A1CRISP-F 5′-TCGTCGGCAGCGTCAGATGTGTATAAGAGACAGcctctctcggcctgacca-3′ and COL6A1CRISP-R 5′-GTCTCGTGGGCTCGGAGATGTGTATAAGAGACAGggcttgttagtgctgtgcaa-3′. Library preparation was carried out as described [27]. Briefly, PCR products were indexed using the Illumina Nextera XT DNA library preparation kit, purified, pooled and sequenced on an MiSeq platform (Illumina, San Diego, CA, USA) using a 2 × 250 paired-end setting. The average depth of coverage was ~20,000× for each sample. The percentages of the different alleles after Cas9-mediated editing were determined using Mosaic Finder bioinformatic software developed in-house [27,28,29,30]. The Mosaic Finder pipeline integrates NGS read mapping, normalization of read counts, variant frequency calculation and genome-editing efficiency statistics at each position of the target region. Briefly, it takes the fastq files generated by pair-end sequencing and generates consensus sequences by joining the corresponding read pairs (forward and reverse). The repertoire of consensus sequences (allelic clusters) represents the allelic diversity generated by the Cas9-mediated edition. These clusters are then aligned against the sequence used as a reference and are classified in allelic classes based on the different types of the identified variant (mismatch, INDEL). The frequency of each cluster is then calculated and plotted.

### 4.8. Digital Droplet PCR

For the amplification of exon 10 wild-type and mutated of *COL6A1*, the forward primer was 5′-CCGGAGATCCTGGAAGA-3′ and the reverse primer was 5′- TTTTTCTCCCTTCATTCCCT-3′. The wild-type allele probe was 5′-CGGGGACCTCGGACC-3′and was 5′ HEX-labeled, and the mutated allele probe was 5′-CGGGGACCTCAGACC-3′ and was 5′ FAM-labeled. ddPCR consisted of the following components (final concentrations in 20 μL total reaction volume): 11 μL ddPCR SuperMix for Probes (no dUTP) (Bio-Rad, Hercules, CA, USA), 450 nM of each primer pair and 250 nM of each probe (for the wild-type allele and the mutated allele). The final volume was adjusted with water to 17 μL. Then, 1.7 μL of 0.015 ng/μL template cDNA (0.025 ng) was added. A total of 20 μL of this mixture was placed into a cell of a Bio-Rad cartridge, and 70 μL of droplet generator oil was added to the well. The cartridge was placed into a QX200 droplet generator (Bio-Rad, Hercules, CA, USA) to generate the droplets. The droplets were then transferred to a 96-well PCR plate. The PCR plate was placed in a thermocycler and amplified with the following cycling conditions: 95 °C for 10 min, 39 cycles of 95 °C for 30 s and 50 °C for 1 min for annealing and extension, 10 min at 98 °C for reaction termination, and cooled to 4 °C. The plate was then placed into the QX200 droplet reader for data analysis. The wild-type and mutant alleles were distinguished by a 2-dimensional view of the ddPCR analysis. For heterozygous mutants, the concentrations of the mutant and the wild-type droplets were analyzed and generated using Bio-Rad QuantaSoftTM software (v1.7.4) with default settings for threshold determination to distinguish positive and negative droplets. At least three biological replicates were carried out for each experiment.

### 4.9. Extracellular Matrix Immunostaining

Skin fibroblast cultures were established and grown as described [12]. Confluent fibroblasts (80–90% confluence) were treated with 25 μg/mL of L-ascorbic acid phosphate magnesium (Wako Chemicals GmbH, Neuss, Germany) for 24 h before fixing in 4% paraformaldehyde for 10 min. The fixed cells were blocked in 3% BSA-phosphate buffered saline (PBS) with 0.05 Tween-20 for 15 min. Then, the cells were incubated with the primary antibody (MAB1944, 1:500 dilution in PBS, Merck Millipore, Billerica, MA, USA) for 1 h at room temperature. After three washes with PBS, the cells were incubated with the secondary antibody (anti-mouse IgG conjugated with Alexa Fluor 488, Thermofisher) diluted 1:500 in a PBS-Tween solution containing DAPI for nuclear staining for 30 min. After this time, cells were washed three times with PBS and preparations were finally mounted using ProLong Diamond mounting medium and stored at 4 °C until viewing.

### 4.10. Confocal and Super-Resolution Imaging

Confocal and super-resolution microscopy analysis was performed on a Leica TCS SP8 microscope equipped with a white light laser, HyVolution and Hybrid spectral detectors (Leica Microsystems GmbH, Mannheim, Germany). The confocal images were acquired using an HC PL APO 20×/0.75 dry immersion objective. The super-resolution images were acquired using an HC PL APO 100×/1.4 oil immersion objective, the HyD detector and HyVolution. DAPI was excited with a blue diode laser (405 nm) and detected at 420–460 nm. COL6 was excited with an argon laser (488 nm) and detected at 500–560 nm. To quantify confocal COL6 images, Z-stacks of 10 sections were acquired every 1.5 μM along the cell thickness (*n* = 3 independent experiments). The variation in the intensity of each spectral component was encoded using 12 bits. Appropriate negative controls were used to adjust confocal settings and avoid non-specific fluorescence artefacts. To compare the confocal data, identical confocal settings were used for image acquisition in different experiments. Image quantification of the integrated density (sum of the intensity of all the pixels in the area delimited by the mask) was performed using ImageJ/Fiji software (NIH, Bethesda, MD, USA). For the super-resolution images (HyVolution mode), the pinhole was set to 0.8 Airy units. To study COL6 distribution in three dimensions, Z-stacks were acquired every 0.35 μM along the cell thickness (35 sections). Image deconvolution was performed with Huygens Professional Software v17.10.0p8 (SVI, Leiden, The Netherlands). COL6 area analysis was performed with Imaris software on 3D super-resolution confocal images. Then, both channels (nuclei and collagen) were reconstructed in 3D using Surface function, and the collagen area was measured using the Surface Statistics function. The same threshold parameters were applied when comparing samples from individuals and healthy controls. To calculate the area of collagen VI used super-resolution stacks, as the fiber structure and their fragmentation in individuals, and the fiber structure recovery after treatment, was more evident at this resolution.

## 5. Conclusions

In conclusion, our work provides a proof-of-principle that a CRISPR/Cas9-based strategy to silence the *COL6A1* c.877G>A; p.Gly293Arg variant in heterozygosity is a promising and effective strategy to restore the molecular and cellular phenotype of fibroblasts. Future work should include in vivo studies in animal models to assess delivery, efficacy and safety, which will be important for clinical translation.

## Data Availability

The data that supported the findings of the present study are available from the corresponding author upon request.

## Supplementary Materials

The following supporting information can be downloaded at: https://www.mdpi.com/article/10.3390/ijms23084410/s1.
