# Supplementary material for: CRISPR/Cas9-Mediated Allele-Specific Disruption of a Dominant COL6A1 Pathogenic Variant Improves Collagen VI Network in Patient Fibroblasts
Source: Int J Mol Sci. 2022 Apr 16;23(8):4410. doi: 10.3390/ijms23084410 (PMC9025481; doi:10.3390/ijms23084410)
Supplement: Supplementary file 1 [file ijms-23-04410-s001.zip › Figure Supplementary 1.pdf]

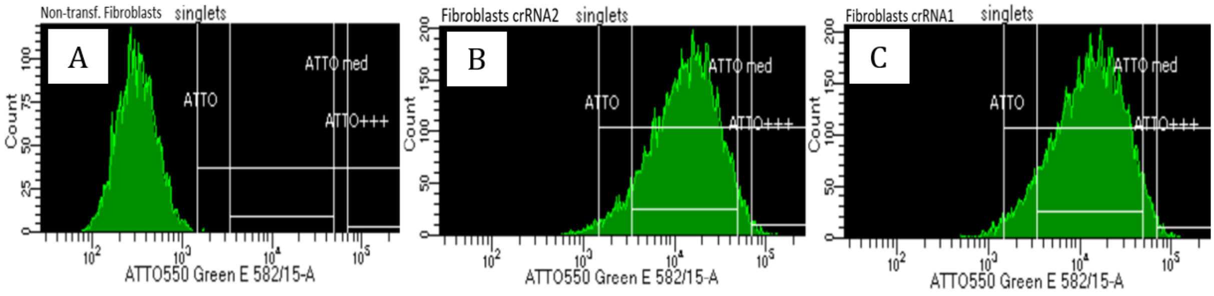

Figure Supplementary 1. Fluorescence Activated Cell Sorting of CRISPR/Cas9-transfected fibroblasts
Emission signals of the ATTO550 fluorophore detected by the flow cytometer corresponding to the transfected fibroblasts from patient 1. A) Non-transfected fibroblasts; B) Fibroblasts transfected with crRNA2 and C) Fibroblasts transfected with crRNA1. This result is representative for all the patients.
