# Supplementary material for: CRISPR/Cas9-Mediated Allele-Specific Disruption of a Dominant COL6A1 Pathogenic Variant Improves Collagen VI Network in Patient Fibroblasts
Source: Int J Mol Sci. 2022 Apr 16;23(8):4410. doi: 10.3390/ijms23084410 (PMC9025481; doi:10.3390/ijms23084410)
Supplement: Supplementary file 1 [file ijms-23-04410-s001.zip › Figure Supplementary 2.pdf]

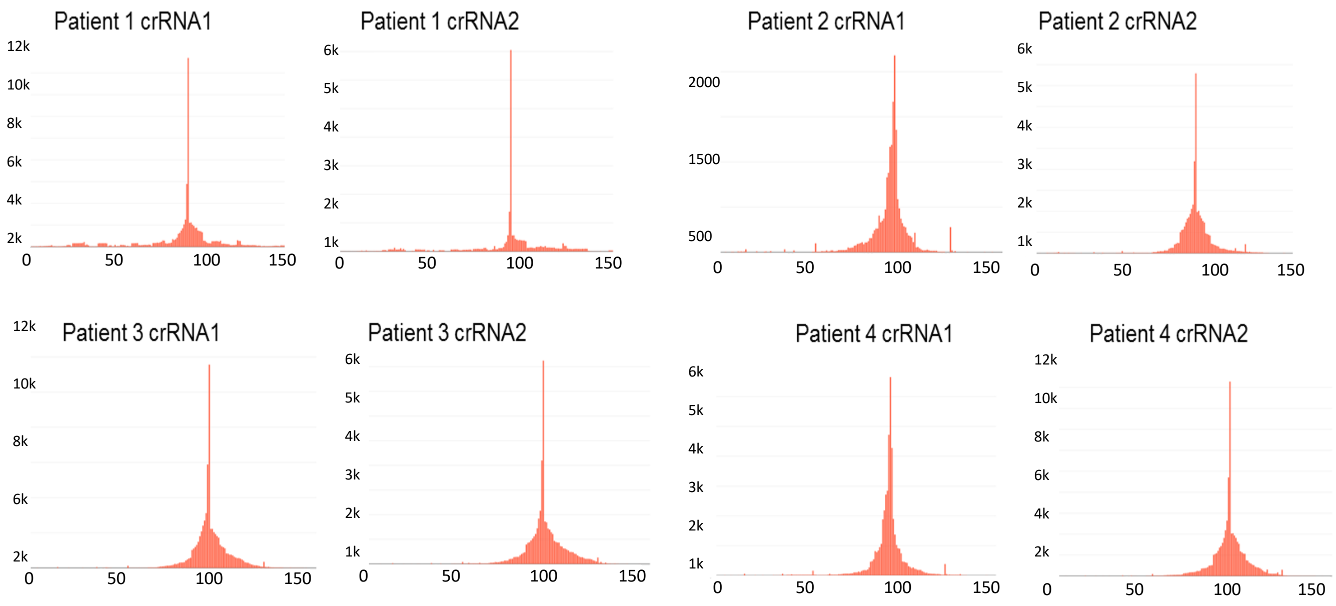

Figure Supplementary 2. Deletion graph of the edited exon 10 of *COL6A1*. Each graph represents the number of individual reads (Y-axis) that a certain deletion has at a certain position (X-axis) of the region of exon 10 of *COL6A1* amplified around the c.877G>A variant for each patient and each guide.
